# Associations Between Circadian Timing and White Matter Microstructure

**DOI:** 10.1002/alz.091538

**Published:** 2025-01-09

**Authors:** Lisa S Weimar, Meina Zhang, Eric Axelson, Vincent A Magnotta, Helen J Burgess, Chooza Moon

**Affiliations:** ^1^ The University of Iowa, Iowa City, IA USA; ^2^ University of Michgan, Ann‐Arbor, MI USA

## Abstract

**Background:**

Individuals with Alzheimer’s disease often exhibit white matter microstructure degenerations observable through diffusion tensor imaging (DTI). The circadian system regulates sleep and wake cycles. As people age, sleep and wake cycles can be disrupted, which can worsen sleep quality. Circadian rhythm disruption can increase the risk of developing Alzheimer’s disease and damage white matter microstructures. However, few studies have investigated the relationship between circadian timing and the structural integrity of white matter among adults.

**Method:**

Data was collected from 49 later‐life adults (51% female) with mean age 70.4 years (SD = 6.50). Diffusion‐weighted MRI sequences were acquired using a 3.0T scanner. Fractional anisotropy (FA) scalar maps were generated through FSL‐DTIFIT, and the Johns Hopkins University‐ICBM white matter atlas was utilized to assess FA values within white matter tracts. Circadian timing was determined by home‐based dim light melatonin onset (DLMO). A t‐test was used to compare FA values by mean DLMO (17: 46).

**Result:**

In this preliminary analysis later DLMO time was associated with greater FA values in the corticospinal (Mean_early DLMO_ = 0.41 (SD = 0.04) vs. Mean _later DLMO_ = 0.44 (SD = 0.04), p = 0.023, uncorrected), right corticospinal (Mean_early DLMO_ = 0.40 (0.05) vs. Mean_later DLMO_ = 0.44 (0.04), p = 0.01, uncorrected), left posterior thalamic radiation (Mean_early DLMO_ = 0.38 (0.03) vs. Mean_later DLMO_ 0.40 (0.03), p = 0.048, uncorrected), cingulum‐hippocampus (Mean_early DLMO_ = 0.26 (0.03) vs. Mean_later DLMO_ = 0.28 (0.03) p = 0.025, uncorrected), and left cingulum‐hippocampus tracts (Mean_early DLMO_ = 0.26 (0.03) vs. Mean_later DLMO_ = 0.29 (0.03) p = 0.01, uncorrected).

**Conclusion:**

This preliminary data demonstrates that abnormally early circadian timing may be associated with reduced white matter integrity in specific tracts. Future research using a larger sample, longitudinal design, and multimodal imaging methods is needed to confirm these findings and identify the mechanisms underlying these associations.

